# The Effects of Operating Room Nurses’ Experiences of Verbal Violence, Resilience, and Social Support on Post-Traumatic Stress

**DOI:** 10.3390/healthcare13162027

**Published:** 2025-08-17

**Authors:** Do Kyun Kim, Sung Hee Shin

**Affiliations:** 1 Department of Nursing, Graduate School, Kyung Hee University, Seoul 02447, Republic of Korea; dogyun1225@khu.ac.kr; 2 College of Nursing Science, Kyung Hee University, Seoul 02447, Republic of Korea

**Keywords:** operating room nurse, post-traumatic stress, verbal violence, resilience, social support

## Abstract

**Background/Objectives:** Operating room (OR) nurses are frequently exposed to high-stress environments, including verbal violence, which may induce post-traumatic stress (PTS). This study investigated the effects of verbal violence, resilience, and social support on PTS among OR nurses. **Methods**: A cross-sectional descriptive correlational study was conducted with 150 OR nurses who had at least one year of work experience. Data were collected via a mobile-based questionnaire and analyzed using SPSS Statistics 25. The main variables included experiences of verbal violence (from doctors and nurses), resilience, social support, and OR work satisfaction. **Results:** Exposure to verbal violence from doctors and nurses, as well as having 10 or more years of clinical experience, were significantly associated with higher PTS levels. In contrast, greater OR work satisfaction was associated with lower PTS. These variables collectively explained 36.6% of PTS variance (F = 8.64, *p* < 0.001). **Conclusions:** Verbal violence significantly contributes to PTS among OR nurses. Enhancing resilience and social support may mitigate the impact of PTS. Therefore, interventions such as structured peer-support systems, resilience training, and violence-prevention policies are recommended to reduce PTS risk. In addition, interventions to prevent verbal violence, and strengthen resilience and social support, and OR work satisfaction are crucial to protect nurses’ mental health and ensure patient safety.

## 1. Introduction

Nurses are known to experience workplace bullying, particularly verbal violence, more frequently than other healthcare professionals. A survey conducted by the Ministry of Health and Welfare in 2021 revealed that nurses experience verbal violence most frequently, accounting for 77.83% of all types of workplace bullying [1]. Operating room (OR) nurses are frequently exposed to verbal violence because of close collaboration with physicians and fellow nurses, which induces significant mental stress [2]. Compared to other hospital departments, the OR environment requires nurses to work closely with doctors, adhere strictly to aseptic techniques, and maintain quick decision-making abilities, demanding high levels of concentration [2,3]. These factors create a tense atmosphere and increase the likelihood of verbal violence [3,4]. Previous studies have indicated that OR nurses in tertiary hospitals experience a relatively high incidence of verbal violence from doctors, possibly due to the frequent verbal instructions issued by physicians in the closed environment of OR [5]. Such verbal violence can lead to emotional issues and decreased trust, adversely impacting patient safety, and if repetitive, can escalate stress levels, potentially resulting in post-traumatic stress (PTS) [4].

PTS is defined as experiencing persistent re-experiencing, avoidance, and hyperarousal symptoms following an extremely traumatic event. Even minor incidents, if repeated, can induce PTS [6]. These symptoms extend beyond individual emotional distress, resulting in nurses’ absenteeism, decreased quality of care, reduced job satisfaction, and diminished job performance, all adversely affecting patient care [7]. Particularly, PTS can endanger patient safety, impair nursing performance because of emotional problems and diminished trust among healthcare providers, and thus, increase the potential for medical accidents [4]. Moreover, it can induce traumatic responses not only in victims but also in observers, creating distrust and conflict within the organization [8]. Additionally, accumulated stress can increase turnover rates, elevate hospital stress management and compensation costs, adversely impact overall hospital operations, and diminish nurses’ quality of life [9]. Furthermore, the closed nature of the OR makes it challenging for victims to escape traumatic incidents, exacerbating emotional stress among nurses [2,3,5,10]. Nonetheless, while PTS has been studied among nurses working in intensive care units or emergency departments [11,12,13], research specifically targeting OR nurses remain scarce, despite their unique exposure to verbal violence in closed and high-stress environments.

PTS levels vary depending on individuals’ perceptions, and resilience has been identified as a crucial personal factor moderating these perceptions [14]. Resilience, defined as the ability to overcome stress and adversity while positively adapting to organizational demands [15], is also regarded as a key determinant of how individuals behave in stressful environments [16]. Prior studies emphasize the importance of resilience research in identifying ways to effectively cope with and grow from stressful or traumatic experiences [16,17]. Furthermore, even when exposed to the same stressful conditions, individuals with higher resilience demonstrate more positive adaptation and recovery compared to others [18]. This resilience significantly impacts nurses’ experience of PTS [11,19]. In addition, social support is considered a major environmental factor in alleviating PTS [20], referring to positive material and emotional assistance individuals receive from their social groups [21]. Furthermore, social support plays a crucial role in mitigating PTS symptoms among nurses and facilitates their continued engagement in clinical practice [22]. However, nurses experiencing PTS may face challenges in communicating effectively with colleagues, which can lead to a perceived lack of adequate support within the workplace [23]. Previous studies have reported that social support significantly influences stress resulting from verbal violence [24] and has a meaningful impact on PTS [25].

OR nurses continuously experience verbal violence from physicians and colleagues within the tense, closed environment of OR, potentially increasing their levels of PTS. Considering the detrimental effects of PTS on nurses’ mental health, there is a clear need to investigate both risk and protective factors associated with PTS in this population. While previous studies have documented workplace violence among nurses, few have specifically examined the combined effects of verbal violence, resilience, and social support on PTS in OR settings. However, they do not receive sufficient attention or support, and relevant studies remain scarce. Therefore, this study aimed to examine the effects of verbal violence, resilience, and social support on post-traumatic stress among OR nurses in South Korea, and to provide foundational data for developing effective strategies to mitigate PTS among OR nurses. Addressing this gap is expected to inform targeted interventions that enhance nurses’ psychological well-being and ensure patient safety.

## 2. Materials and Methods

### 2.1. Research Design

A cross-sectional descriptive correlational study was conducted to assess the association between verbal violence, resilience, social support, and PTS among OR nurses.

### 2.2. Participants

The participants included OR nurses with at least one year of clinical experience in either circulating or scrubbing roles, working at tertiary and general hospitals in the Seoul and Gyeonggi regions of South Korea. Nurses who were on leave or employed in part-time positions were excluded from the study.

Sample size was calculated using the G*Power 3.1.9.7 software based on multiple regression analysis. Referring to a previous study [26], the required sample size was determined to be 131, with an effect size (f) of 0.15, significance level (α) of 0.05, power (1−β) of 0.80, and 13 predictor variables. To account for dropout, the target sample size was set at 146. Ultimately, 150 participants were recruited through convenience sampling.

### 2.3. Instruments

A structured questionnaire was used, comprising 82 items covering post-traumatic stress (22), experiences of verbal violence (17), resilience (25), social support (8), and general characteristics (10). All instruments were used with permission from the original developers.

#### 2.3.1. Post-Traumatic Stress

PTS was measured using the Korean version of the Impact of Event Scale-Revised (IES-R). It was translated by Eun et al. [27] from the original by Weiss and Marmar [28]. It consists of 22 items across four subscales and uses a 5-point Likert scale. Total scores range from 0 to 88; higher scores indicate greater severity. Cronbach’s α was 0.94 in this study.

#### 2.3.2. Experience of Verbal Violence

This was assessed based on Nam et al.’s tool [4], which includes separate subscales for violence from physicians (10 items) and nurses (7 items), rated on a 4-point Likert scale. Verbal violence is conceptually defined as violent speech that demeans or humiliates others, unjustifiably hurting their feelings or violating their dignity [4]. Cronbach’s α in this study was 0.88 overall, with α = 0.84 for each subscale.

#### 2.3.3. Resilience

Resilience was measured using the Korean version of the Connor–Davidson Resilience Scale (CD-RISC) by Baek [29]. It comprises 25 items that are rated on a 5-point Likert scale. Higher scores indicate greater resilience. Cronbach’s α was 0.90 in this study.

#### 2.3.4. Social Support

Social support was assessed using the translated version of a tool developed by House [30]. The translation was performed by Son and Ko [31]. This study focused on support from supervisors and colleagues—four items each, rated on a 5-point Likert scale. Cronbach’s α in this study was 0.78 overall.

#### 2.3.5. General Characteristics

These were assessed via 10 items including hospital type, age, gender, educational level, marital status, clinical experience, position, satisfaction with OR work, incident response, and disciplinary regulations. Satisfaction was measured on a scale from 0 to 10 and categorized into low (0–3), moderate (4–7), and high (8–10). Cronbach’s α was 0.82.

### 2.4. Data Collection and Ethical Considerations

This study was approved by the Institutional Review Board of S Hospital (SMC2023-07-064-003). Data were collected via a self-administered mobile survey (Google Forms) between 28 August and 8 September 2023, distributed through MyDuty (an app for South Korean nurses). To ensure that only OR nurses participated, the eligibility criteria were clearly highlighted in bold on the informed consent form. The electronic survey system required participants to provide consent before they could access the questionnaire, and they were informed that they could withdraw at any time if they disagreed with any item. Convenience sampling was employed, and a total of 150 OR nurses voluntarily and anonymously completed the survey. There were no incomplete responses, so all questionnaires were included in the final analysis. All responses and personal data were used solely for research purposes, numerically coded, securely stored on an encrypted, password-protected computer accessible only to the researchers, and will be permanently destroyed three years after study completion. A digital gift voucher was provided as an incentive, with related personal data deleted immediately after distribution.

### 2.5. Data Analysis

Data were analyzed using SPSS Statistics version 25. Descriptive statistics summarized participants’ characteristics and study variables. T-tests and one-way ANOVA were performed to assess differences in PTS; Scheffé’s test was conducted post hoc. Reliability was evaluated using Cronbach’s α. Pearson’s correlation was employed to assess variable relationships. Multiple regression analysis using the enter method was applied to identify predictors of PTS.

## 3. Results

### 3.1. General Characteristics of Participants and Differences in Post-Traumatic Stress According to General Characteristics

The general characteristics of the participants and differences in PTS based on these characteristics are summarized in Table 1. A majority of the participants (69.4%) were employed at tertiary hospitals, and most were female (85.4%). The most common age group was 26–30 years (40.7%), and 90.0% had completed a four-year university education. Regarding marital status, 76.0% were single. In terms of clinical experience, 34.6% had between five and less than 10 years of experience. Most participants (90.6%) were staff nurses. Regarding satisfaction, 56.0% reported moderate satisfaction with OR work, 42.6% reported moderate satisfaction with hospital response to incidents, and 44.0% reported low satisfaction with disciplinary regulations.

Significant differences in PTS scores were observed by gender, age, clinical experience, and satisfaction variables. Male nurses reported significantly higher PTS scores (41.68) compared to female nurses (31.86) (t = 2.19, *p* = 0.03). Age also showed a significant difference (F = 3.48, *p* = 0.02); participants aged ≥36 years had higher scores (43.48) than those aged ≤25 (23.98), with the 26–30 (32.38) and 31–35 (31.78) age groups showing intermediate values. Participants with ≥10 years (42.18) and 5–<10 years (36.27) of clinical experience reported higher PTS compared to those with <3 years (30.29) and 3–<5 years (26.88) (F = 4.57, *p* = 0.01). Those with low satisfaction regarding OR work (44.83) had significantly higher PTS than those with moderate (33.16) or high (27.94) satisfaction (F = 6.07, *p* = 0.01). Similar trends were observed for satisfaction with hospital response (F = 8.01, *p* < 0.01) and disciplinary regulations (F = 3.25, *p* = 0.04). No significant differences in PTS were identified based on hospital type, education level, marital status, or job position.

### 3.2. Levels of Verbal Violence Experience, Resilience, Social Support, and Post-Traumatic Stress

The levels of verbal violence, resilience, social support, and PTS are presented in Table 2. The mean PTS score was 1.58 ± 0.90 (out of 4). Mean scores for verbal violence were 2.08 ± 0.58 for violence from doctors and 1.94 ± 0.65 for violence from nurses. Resilience had a mean of 2.45 ± 0.59, and social support scored 3.18 ± 0.72. Skewness and kurtosis values for all variables were within acceptable limits (±2 and ±7, respectively), confirming normality. Thus, parametric statistical analyses, including multiple regression, were considered appropriate.

### 3.3. Correlations Among Verbal Violence, Resilience, Social Support, and Post-Traumatic Stress

Pearson’s correlation coefficients are summarized in Table 3. Verbal violence from doctors (r = 0.51, *p* < 0.001) and from nurses (r = 0.44, *p* < 0.001) were positively correlated with PTS. Resilience (r = −0.28, *p* < 0.001) and social support (r = −0.23, *p* = 0.004) were negatively correlated with PTS.

### 3.4. Factors Influencing Post-Traumatic Stress

Multiple regression analysis results are presented in Table 4. Demographic variables (age, gender, clinical experience), satisfaction levels (with OR work, hospital response, disciplinary regulations), and key psychological variables (resilience, social support, verbal violence) were included. Dummy variables were constructed for age (≤30 as reference), gender (female as reference), and clinical experience (<3 years as reference). The Durbin–Watson statistic was 1.94, indicating no autocorrelation. Tolerance values were >0.1 and variance inflation factors (VIFs) <10, suggesting no multicollinearity.

The final model explained 36.6% of the variance in PTS (F = 8.64, *p* < 0.001), which aligns with explanatory power levels commonly reported in psychosocial and nursing research [32]. Verbal violence from doctors (β = 0.31, *p* < 0.01), from nurses (β = 0.31, *p* < 0.01), and ≥10 years of clinical experience (β = 0.36, *p* = 0.01) significantly increased PTS. In contrast, higher satisfaction with OR work was negatively associated with PTS (β = −0.17, *p* = 0.04).

## 4. Discussion

Based on the findings of this study, several important implications regarding post-traumatic stress (PTS) among OR nurses can be drawn. The average PTS score among participants was 34.8, which exceeds the post-traumatic stress disorder (PTSD) screening threshold of 25 suggested in a previous study [33]. This indicates a clinically concerning level of psychological distress. PTS has been linked to declining job satisfaction, diminished productivity, and an increased likelihood of nursing errors [10], ultimately compromising care quality and patient safety. Therefore, systematic screening and counseling systems should be established to monitor PTS levels and enable early organizational intervention.

Notably, this study identified that verbal violence was more frequently experienced from doctors than from nurses, which is consistent with the results of previous research [2,3,5]. Previous studies have highlighted that operating rooms are closed, high-pressure environments characterized by hierarchical communication and authority-centered culture—key contributors to verbal violence [2,3]. These structural dynamics may amplify verbal abuse directed from doctors to nurses, intensifying emotional exhaustion. To mitigate verbal violence, organizations must reinforce management systems, implement awareness and communication training, and adopt effective violence prevention programs.

In this study, resilience and social support were not found to have a statistically significant effect on PTS. Prior research specifically examining these factors in relation to PTS among OR nurses are limited, making direct comparisons difficult. However, several recent studies support the protective role of resilience and social support against PTS and related psychological distress among nurses. Kim et al. [34] reported that Korean hospital nurses with lower levels of resilience and social support were more likely to be classified in a high-risk PTSD group. Similarly, Katsiroumpa et al. [35] demonstrated that resilience and social support significantly alleviate symptoms of anxiety and depression, suggesting their potential contribution to mitigating PTSD symptoms. These findings suggest that both are stable psychological resources [23] that can be enhanced through structured support, education, and training [18]. As resilience can be improved, resilience-building workshops and psychoeducational programs should be implemented. To bolster social support, institutions should create peer support programs, facilitate regular feedback between junior and senior nurses, and promote a culture of mutual respect.

Significant differences in PTS were observed based on gender, age, clinical experience, OR work satisfaction, hospital response satisfaction, and disciplinary regulation satisfaction. Male nurses reported higher PTS levels than female nurses. This may stem from factors such as higher occupational stress levels observed among male nurses compared to their female counterparts, as reported in previous research [36]. Therefore, career coaching and gender-sensitivity training should be introduced to support professional identity and address workplace stereotypes.

Furthermore, the finding that nurses with greater clinical experience had higher PTS levels suggests that seasoned nurses—often the backbone of hospital operations—are chronically exposed to stress. This may lead to long-term workforce attrition. Although there is limited prior research directly examining why more experienced nurses report higher levels of PTS, making direct comparisons difficult. It is plausible that experienced OR nurses are repeatedly exposed over their careers to verbal violence, critical incidents, and high-pressure situations. Prior research has shown that both direct and indirect chronic exposure to workplace violence significantly increases the risk of psychological disorders related to PTSD, particularly when multiple forms of violence and repeated stressors coexist [37]. Consequently, consistent with previous findings [12], experienced nurses’ departure due to stress is a serious concern. Hospitals should implement systematic stress management strategies, including regular psychological assessments, counseling services, and post-trauma education. Supporting the emotional health of experienced nurses not only enhances their well-being but also contributes to staff retention and care continuity.

This study confirmed that verbal violence was positively correlated with PTS, while resilience and social support were negatively correlated. These results align with the results of previous studies [11,38,39], emphasizing the role of resilience in coping with PTS [38] and social support as a protective buffer [39]. Thus, both personal resources (resilience) and organizational resources (social support) must be considered to mitigate PTS [23]. Peer networks, mentoring, and other initiatives that foster emotional support should be prioritized to alleviate psychological burdens and promote sustainable nursing practices.

The multiple regression analysis revealed key factors affecting PTS. Higher verbal violence from both doctors and nurses, ≥10 years of clinical experience, and low satisfaction with OR work was significantly associated with elevated PTS. These results are in line with previous findings [13], highlighting that verbal violence is not just unpleasant but can induce lasting trauma.

Particularly, the high PTS levels in experienced nurses support earlier findings linking stress in long-tenured staff with increased turnover risk [40]. This poses a serious threat to workforce stability. The association between lower OR work satisfaction and higher PTS also aligns with the findings of previous studies [41] and may reflect cumulative emotional exhaustion.

Improving the OR work environment requires fundamental adjustments. Structural interventions, such as communication improvements and role redesign, are necessary. Enhancing work satisfaction may be an effective strategy to reduce PTS. Accordingly, hospitals should develop standardized response protocols and implement comprehensive policies to systematically manage PTS.

This study is crucial mainly because it identified key factors contributing to PTS and provides practical evidence for developing mitigation strategies. Future research should also incorporate qualitative approaches to gain deeper insights into the lived experiences of OR nurses. Moreover, this study serves as a foundation for applying more advanced statistical methods in future investigations to further refine understanding of PTS-related factors.

As this study was conducted with nurses in tertiary and general hospitals in Seoul and Gyeonggi, the generalizability of the findings may be limited. Nurses from specialized surgical hospitals were not included. Furthermore, this study relied on convenience sampling from a limited geographic region, which may not represent the broader population of OR nurses across South Korea. Self-reported measures were used to assess exposure to verbal violence, resilience, social support, and PTS, which may introduce recall bias or social desirability bias. Although validated instruments were employed, their validity and reliability could vary depending on the cultural and organizational context, and this was not examined in detail. Future studies should broaden the scope to include more regions and hospital types and employ more diverse data collection methods.

Hospitals may use these findings as foundational evidence when conducting regular surveys or developing intervention programs to prevent verbal violence and alleviate stress among OR nurses.

## 5. Conclusions

This study identified verbal violence, long clinical experience, and low job satisfaction as key factors contributing to PTS among OR nurses. These findings highlight the urgent need for institution-level interventions that actively prevent verbal violence, provide comprehensive psychological support, and foster a safe, respectful workplace culture. Addressing PTS in this population is not only essential for protecting OR nurses’ mental health but also a critical component of ensuring patient safety and sustaining the quality of surgical care.

## Figures and Tables

**Table 1:** General Characteristics and the Differences in Post-Traumatic Stress According to the General Characteristics of Operating Room Nurses (*N* = 150).

Characteristics	Categories	N	%	Post-Traumatic Stress				
				M	SD	t/F	*p*	Scheffe
Hospital type	Advanced General hospital	104	69.4	34.83	19.45	1.46	0.15	
	General hospital	46	30.6	30.02	20.40			
Sex	Male	22	14.6	41.68	18.14	2.19	0.03	
	Female	128	85.4	31.86	19.83			
Age (yr)	≤25 ^a^	13	8.6	23.92	18.50	3.48	0.02	a < b, c < d
	26~30 ^b^	61	40.7	32.38	19.14			
	31~35 ^c^	51	34.0	31.78	18.89			
	≥36 ^d^	25	16.7	43.48	21.60			
Education	Associate degree	8	5.4	40.63	21.13	0.62	0.54	
	Bachelor’s degree	135	90.0	32.90	19.60			
	Master’s degree	7	4.6	31.00	24.79			
Marital status	Unmarried	114	76.0	31.92	18.99	−1.53	0.13	
	Married	36	24.0	37.64	22.26			
Work experience (yr)	1~<3 ^a^	27	18.0	30.29	17.49	4.57	0.01	a, b < c, d
	3~<5 ^b^	44	29.4	26.88	21.54			
	5~<10 ^c^	52	34.6	36.27	16.39			
	≥10 ^d^	27	18.0	42.18	21.03			
Position	General nurse	136	90.6	33.37	19.65	0.32	0.75	
	Charge nurse or higher	14	9.4	31.67	22.34			
Operating room work satisfaction	High (8~10) ^a^	43	28.6	27.94	21.73	6.07	0.01	a, b < c
	Middle (4~7) ^b^	84	56.0	33.16	18.24			
	Low (0~3) ^c^	23	15.4	44.83	17.02			
Hospital response satisfaction	High (8~10) ^a^	24	16.0	21.44	21.83	8.01	<0.001	a < b, c
	Middle (4~7) ^b^	64	42.6	34.77	17.93			
	Low (0~3)^c^	62	41.4	37.61	18.61			
Punishment regulation satisfaction	High (8~10) ^a^	30	20.0	30.03	20.78	3.25	0.04	a, b < c
	Middle (4~7) ^b^	54	36.0	29.83	20.24			
	Low (0~3) ^c^	66	44.0	37.91	18.26			

M: mean, SD: standard deviation.

**Table 2:** Level of Post-Traumatic Stress, Experience of Verbal Violence, Resilience, and Social Support (*N* = 150).

Variables	Minimum	Maximum	Mean	SD	Skewness	Kurtosis
Post-traumatic stress	0	4	1.58	0.90	−0.12	−0.90
Experience of verbal violence from doctor	1	4	2.08	0.58	0.27	−0.31
Experience of verbal violence from nurse	1	4	1.94	0.65	0.65	0.10
Resilience	0	4	2.45	0.59	0.18	−0.58
Social support	1	5	3.18	0.72	0.01	−0.63

SD: standard deviation.

**Table 3:** Correlation among Experience of Verbal Violence, Resilience, Social Support, and Post-Traumatic Stress (*N* = 150).

Variables	1 r(*p*)	2 r(*p*)	3 r(*p*)	4 r(*p*)	5 r(*p*)
1. Experience of verbal violence from doctor	1				
2. Experience of verbal violence from nurse	0.54 (<0.001)	1			
3. Resilience	−0.45 (<0.001)	−0.41 (<0.001)	1		
4. Social support	−0.24 (0.002)	−0.21 (0.007)	0.52 (<0.001)	1	
5. Post-traumatic stress	0.51 (<0.001)	0.44 (<0.001)	−0.28 (<0.001)	−0.23 (0.004)	1

**Table 4:** The Effects of Operating Room Nurses’ Experiences of Verbal Violence, Resilience, and Social Support on Post-Traumatic Stress (*N* = 150).

Characteristics	B	SE	β	t	*p*
Experience of verbal violence from doctor	0.48	0.13	0.31	3.74	<0.01
Experience of verbal violence from nurse	0.43	0.12	0.31	3.68	<0.01
Resilience	0.03	0.13	0.02	0.24	0.81
Social support	−0.01	0.10	−0.01	−0.11	0.91
Clinical experience (yr) (ref = 1~<3)					
3~<5	−0.03	0.18	−0.07	−0.18	0.86
5~<10	0.23	0.22	0.12	1.04	0.30
≥10	0.86	0.27	0.36	3.21	0.01
Age (yr) (ref = ≤30)					
≥31	−0.19	0.17	−0.11	−1.11	0.27
Sex					
Male (ref = Female)	0.23	0.18	0.09	1.27	0.21
Operating room work satisfaction	−0.08	0.04	−0.17	−2.13	0.04
Hospital response satisfaction	−0.03	0.04	−0.07	−0.59	0.56
Punishment regulation satisfaction	0.01	0.04	0.03	0.26	0.80
F (*ρ*)	8.64 (<0.001)				
Adj *R*^2^	0.366				

SE = standard error; Ref = reference category.

## Data Availability

The raw data supporting the conclusions of this article will be made available by the authors upon reasonable request after signing a confidentiality agreement.
